# Engaging communities to inform the development of a diverse cohort of cancer survivors: formative research for the eat move sleep study (EMOVES)

**DOI:** 10.1186/s40900-023-00529-z

**Published:** 2023-12-11

**Authors:** Ghilamichael Andemeskel, Nynikka R. Palmer, Rena Pasick, Erin L. Van Blarigan, Stacey A. Kenfield, Rebecca E. Graff, Michael Shaw, Wil Yu, Mayte Sanchez, Roberto Hernandez, Samuel L. Washington, Salma Shariff-Marco, Kim F. Rhoads, June M. Chan

**Affiliations:** 1 Helen Diller Family Comprehensive Cancer Center, Office of Community Engagement, University of California, San Francisco, CA USA; 2 Department of Urology, University of California, San Francisco, CA USA; 3 Department of Radiation Oncology, University of California, San Francisco, CA USA; 4 School of Medicine, University of California, San Francisco, CA USA; 5 Department of Epidemiology and Biostatistics, University of California, San Francisco, CA USA; 6 Community Advisory Board, Office of Community Engagement, University of California, San Francisco, CA USA; 7 Men’s Health Committee, Office of Community Engagement, University of California, San Francisco, CA USA; 8 California Health Plan, Common Wealth Care Alliance, San Francisco, CA USA; 9 Cancer Resource Centers of Mendocino County, Ukiah, CA USA; 10 CANA Cultura y Arte Nativa de Las Americas, San Francisco, CA USA

## Abstract

**Background:**

There are more than 18 million cancer survivors in the United States. Yet, survivors of color remain under-represented in cancer survivorship research (Saltzman et al. in Contemp Clin Trials Commun 29:100986, 2022; Pang et al. in J Clin Oncol 34:3992–3999, 2016; Lythgoe et al. in Prostate Cancer Prostatic Dis 24:1208–1211, 2021). Our long-term goal is to enroll and follow a cohort of historically under-represented cancer survivors, to better understand modifiable risk factors that influence clinical and quality of life outcomes in these populations. Towards that goal, we describe herein how we applied community-based participatory research approaches to develop inclusive study materials for enrolling such a cohort.

**Methods:**

We implemented community engagement strategies to inform and enhance the study website and recruitment materials for this cohort including: hiring a dedicated engagement coordinator/community health educator as a member of our team; working with the Helen Diller Family Comprehensive Cancer Center Office of Community Engagement (OCE) and Community Advisory Board members; presenting our educational, research, and study recruitment materials at community events; and establishing a community advisory group specifically for the study (4 individuals). In parallel with these efforts, 20 semi-structured user testing interviews were conducted with diverse cancer survivors to inform the look, feel, and usability of the study website.

**Results:**

Engagement with community members was a powerful and important approach for this study’s development. Feedback was solicited and used to inform decisions regarding the study name (eat move sleep, EMOVES), logo, study website content and imagery, and recruitment materials. Based on community feedback, we developed additional educational materials on healthy groceries and portion size in multiple languages and created a study video.

**Conclusions:**

Including an engagement coordinator as a permanent team member, partnering with the institutional community outreach and engagement resources (i.e., OCE), and allocating dedicated time and financial support for cultivating relationships with stakeholders outside the university were critical to the development of the study website and materials. Our community guided strategies will be tested as we conduct enrollment through community advisor networks and via the state cancer registry.

**Supplementary Information:**

The online version contains supplementary material available at 10.1186/s40900-023-00529-z.

## Abstract (plain language summary)

Under-represented racial and ethnic populations are diagnosed with and die from cancer at higher rates than white Americans but are less likely to be included in research studies. This has resulted in limited data on these populations, especially regarding cancer survivorship and lifestyle factors such as diet, exercise, and sleep. Our aim was to develop inclusive and appealing study materials for enrolling a diverse cancer survivorship cohort by integrating a community engagement coordinator/health educator into the research team and collaborating with our cancer center’s office of community engagement community advisory board. An additional bridge was developed between community partners and the research team by establishing a community advisory board specifically for the study. We also conducted 20 user testing interviews with cancer survivors and community stakeholders to inform the look, feel, and usability of the study website during development. Our community partnerships and interviews assisted with decisions on our study name, Eat Move Sleep Study (EMOVES), logo, redesigning the study website, and study format. Our partners also provided guidance that highlighted community need and development of new educational materials for healthy diet (postcard sized grocery list on healthy eating) and a video-based recruitment tool for the study. Incorporation of an engagement coordinator into the research team, building an ongoing relationship with our cancer center’s office of community engagement, and adding community advisors onto our study team has greatly impacted our study approach and design.

**Supplementary Information:**

The online version contains supplementary material available at 10.1186/s40900-023-00529-z.

## Introduction

In the United States, an estimated 40–45% of cancer diagnoses and deaths are preventable through modifying health behaviors such as tobacco cessation, limiting UV exposure, regular physical activity, maintaining a healthy diet, and weight management [4]. There is less research specifically addressing *if and how* such practices improve survivorship outcomes *after* diagnosis, though reports suggest these modifiable factors can lead to improvements in clinical outcomes [5–7]. However, there are limitations to these studies; people who self-identify as Black, Latinx, American Indian/Alaska Native, Asian American, or Native Hawaiian/Pacific Islander, who harbor the greatest burden of disease, are under-represented in cancer survivorship studies [1–3, 8, 9] and in the limited existing studies addressing health behaviors and cancer progression and death outcomes [10–17]. Under-representation of these populations in epidemiologic studies hinders the ability of researchers, policymakers, and practitioners to reduce or eliminate cancer health disparities.

To address this lack of diversity and inform the best sustainable prevention and treatment strategies to support these vulnerable populations, our long-term goal is to enroll and follow a cohort of racially, ethnically, geographically, and socioeconomically diverse cancer survivors, collecting data on social and financial needs, health habits, integrative medicine, quality of life, and sociodemographic and clinical factors, using a digital research platform. Our short-term goal, as described herein, was to apply community-based participatory research approaches to develop inclusive and appealing study materials for enrolling such a cohort, with an initial focus on prostate, colorectal, and bladder cancer survivors [18]. This included integrating a community engagement coordinator/health educator, partnering with our cancer center’s office of community engagement (OCE) community advisory board (CAB), engaging community representatives, and centering the development of the study website and materials around community feedback and needs. This report summarizes the community engagement strategies, actions taken in response to community feedback, and findings from user testing interviews that guided the development of the study website and recruitment materials for the Eat Move Sleep (EMOVES) pilot cohort.

## Materials and methods

### Community engagement overview

Our approach for the initial design of this study was influenced by four key principles of community-based participatory research from Israel BA et al.: “Builds on strengths and resources within the community;" "Facilitates collaborative partnerships in all phases of the research"; "Involves a cyclical and iterative process"; and "Disseminates findings and knowledge gained to all partners" [19]. To enact these principles, our team included an engagement coordinator/community health educator, the UCSF Helen Diller Family Comprehensive Cancer Center (HDFCCC) Office for Community Engagement (OCE), and the Community Advisory Board (CAB) of the OCE, and we established a study-specific CAB. With the support of these partnerships, we also conducted user testing interviews to inform the development of the study website. Each of these critical partners and the methods for our interviews are described below. Additional reporting on patient and public involvement is included using the Guidance for Reporting Involvement of Patients and the Public (GRIPP2) Long-Form (Supplemental Methods) [20].

#### Dedicated engagement coordinator

In acknowledgement that social concordance (e.g., racial and ethnic group, gender, etc.) of health professionals/study teams with patients and potential study participants can increase care satisfaction and study enrollment, respectively [21–28], we integrated a dedicated engagement coordinator/community health educator (GA) into our research team who functioned both as a community liaison and research coordinator (25% full-time equivalent) for studies focused on prostate cancer. This role was developed in 2018 for an established community member with experience in trust-building, networking, and needs assessment/delivery, specifically with Black communities in the San Francisco Bay Area (given the focus of initial pilot funding, see below). Our new team member contributed prior experience in research and working with community organizations that supported Black communities in the Greater San Francisco Bay Area. The coordinator regularly attended local events of community stakeholders and attended bi-weekly research meetings with the study team. This role was formally expanded and integrated into the HDFCCC OCE in 2019 as a community health educator position, funded by a supplement to the Cancer Center Support Grant (supplement P0535514 to P30CA082103), whereby the individual also took on additional projects for one other principal investigator (PI). The integrated role supported a portfolio of research and education for two PIs and the OCE, all with a focus on health disparities, community engagement, and/or cancer prevention and survivorship.

#### UCSF HDFCCC Office of Community Engagement and Community Advisory Boards

Community outreach and engagement (COE) is “a fundamental activity of National Cancer Institute (NCI)-designated Cancer Centers” [29]. At UCSF, the HDFCCC OCE (led by co-author KFR) is the central hub for community outreach and engagement activity [30]. The OCE’s mission is to “eliminate the inequities that cause cancer disparities in the HDFCCC catchment area by sustaining year-round non-transactional community engagement, facilitating community-academic partnerships for research and service, and disseminating cancer information to increase awareness and knowledge.” Towards these goals, the OCE serves as a bridge between institution, researcher, and community. It maintains these relationships, in part, by convening a Community Advisory Board (CAB) that consists of community stakeholders, and a quarterly lecture series titled “CAB2 Chat-n-Chew,” where researchers present their work to both CAB and community members for feedback (hereafter, we refer to the “OCE CAB” as indicating either of these two CAB groups).

#### Dedicated Community Advisory Group for EMOVES

In Spring 2020, co-author KFR, in her role as the Director of the OCE, reached out to a network of community stakeholders affiliated with the OCE CAB to assess interest in serving as EMOVES advisors. We sought a diverse group of advisors including Black, Latinx, and Asian American/Pacific Islander (AAPI) members, as well as rural residents and those of lower socioeconomic status (SES). Based on response to an email invitation from Dr. Rhoads, four community advisors were identified and joined the study team, collectively representing each of these sociodemographic perspectives (i.e., co-authors M Shaw for Black population, R Hernandez for Latinx community, W Yu for AAPI populations, and M Molina-Saucedo for rural communities). All indicated that their respective communities include those of lower SES. These individuals comprise our EMOVES community advisory group (distinct from the OCE CAB groups) and have provided feedback on study logo selection, advertising materials, the website, and grant applications to sustain and expand EMOVES. While additional grant funding is pending, the group has been compensated by the OCE at the standard UCSF OCE rate of $125/h for community advisors. This allows for the research team to maintain a consistent partnership with community facilitated by the OCE, which works to maintain long-term relationships.

### Methods for interviews

In addition to input from OCE CAB and the EMOVES community advisory group, we sought first-hand input from community members through user testing interviews focused on the look, feel, and usability of the EMOVES study website. Specifically, we sought perspectives on how to enhance and convey the use and value of the study for the under-represented populations, whom we wished to encourage to join. First, we interviewed 10 cancer survivors when we were making early decisions regarding study name, study logo, and look and feel of the website (via static PDF mock-ups, including four public-facing pages on study overview, team, frequently asked questions, and contact-us webpages) (Phase I). We conducted an additional 10 interviews with new participants for after the initial technical build of the website, when participants could trial the beta version that included the four public-facing webpages on study background; four online consent pages (i.e., introduction to consent, main study consent via DocuSign, consent for future research contact, and consent for urology outcomes database for UCSF Dept. of Urology patients only); and post-consent webpages for baseline survey administration (15 survey modules with 7 images) (Phase II).

Eligible individuals included adults over the age of 18, with or without cancer, who were English-speaking, and had access to the Internet, with a focus on under-represented people broadly; a priori, we oversampled for Black men given the stark prostate cancer mortality disparity experienced by this group and initial pilot funding focused thematically on prostate cancer. Interview participants were identified through advertising at local community health events and with the help of CAB members utilizing their network (conducted by the team’s engagement coordinator, GA); investigator announcements at three cancer patient-oriented conferences and presentations at three local prostate cancer support groups (two general support groups in Marin and San Francisco, and one for Black men with prostate cancer in Oakland); and 21 referrals from open studies for cancer patients in the UCSF Depts. of Urology and Medicine.

Interviews of approximately 30–45 min in length were conducted in-person (4 sessions; 6 people) or via Zoom (12 sessions; 14 people). Participants were presented with the aforementioned study materials. Interviews were conducted with one participant, study interviewer and, when available, our website platform developer. We initially sought to conduct group interviews, however due to logistical challenges of scheduling, converted to 1:1 interviews on zoom. Group interviews were conducted on three occasions, two sets of in-person of couples whose partners were their caregivers assisting with technology needs (pre-pandemic), and one group of three individuals via zoom; all other interviews were conducted on zoom to accommodate pandemic restrictions.

The study interviewer (GA) followed a standard set of questions focusing on the look, feel, and usability of the website (see Additional file 1: Methods), and participants were also encouraged to offer their broad input. Interviews were not recorded; the interviewer recorded responses in a Google sheet. All comments and suggestions were noted and discussed with investigators. Notes from the interviews were tracked and categorized in Excel into the following themes/content areas: esthetics (color, layout, font, and images), phrasing, clarity, and website navigation/usability at first by a member of the website design team then by a single interviewer. These were shared with the broader study team to guide study design decisions and next steps.

All research was approved by the Human Research Protection Program’s Institutional Review Board at UCSF.

## Results

Below we present the results of the various community-engaged approaches the study team took to solicit community feedback from and cultivate relationships with community stakeholders. These included regular non-transactional participation and visibility of the community engagement coordinator/study team at community events, presentations to the OCE CAB, seeking input from the EMOVES community advisory group, and semi-structured user testing interviews.

### Engagement coordinator activities

The coordinator regularly attended local events/meeting, ~ 46 annually reoccurring events/meeting and 1–2 monthly events on request (Table 1) to understand the priorities of and to nurture long-term relationships with local community health-focused stakeholders. Given the intermittent nature of research development, the coordinator would regularly attend community events to provide evidence-based diet/exercise resources (e.g., educational booklets or postcards) on behalf of the team to raise/maintain awareness and visibility, and engage interest in cancer-related studies that were open or recruiting. In regular meetings with the study team, the coordinator shared community feedback, including suggestions for the study and comments on educational materials.

**Table 1:** Community network and event engagement

Partners/event	1st engagement	Frequency	Type
Prostate Health Support Group for African American Men (CAB Men’s Health Committee and SFCAN, The San Francisco Cancer Initiative^a^)	7/3/18	Every 1st and 3rd Tuesday of the month	Community based support group
SFCAN Prostate Cancer Action Network (PCAN)	7/6/18	Every first Friday (currently on need/request basis)	SF/UCSF community-based coalition
Faith Communities Committee	9/12/18	On need/request basis	UCSF OCE CAB sub committee
CAB Men’s Health Committee	9/24/18	Every second Thursday	UCSF OCE CAB sub committee
UCSF/California Prostate Cancer Coalition Patient Symposium	6/8/2019	Annual	Educational conference
UCSF Patient Services Committee	7/3/19	On need/request basis	UCSF Cancer center patient committee
Glad Tiding Men’s Health Symposium	7/19/19	Annual event	Health Symposium
Soul Stroll	8/18/19	Annual event	Health Walk/Community Screening
California Prostate Awareness Coalition Support Group Leadership workshop	11/21/19	Annual event	Workshop

*PCa* prostate cancer, *SFCAN* San Francisco Cancer Action network, *PCAN* prostate action cancer network, *UCSF* University of California San Francisco, *OCE* Office of Community Engagement, *CAB* Community Advisory Board

Reference: ^a^Hiatt RA, Sibley A, Venkatesh B, et al. From Cancer Epidemiology to Policy and Practice: The Role of a Comprehensive Cancer Center. *Curr Epidemiol Rep.* 2022;9(1):10–21. PMC8935108

### Presentations from Research Team at Community Events

Over a 14-month period we presented information about our research team and open/enrolling studies at community events (summarized in Tables 1 and 2). The team’s engagement coordinator was present at all events in Table 1 and was joined by study team investigators who presented, listened to feedback, and answered questions at four OCE CAB, three patient support group, and two EMOVES community advisory group meetings.

**Table 2:** Community interaction to develop the eat move sleep study, by year

2018	Hired community outreach coordinator (GA) Submitted early proposal for pilot funding on UCSF open proposal system (*Diversifying Electronic Cohort Research at UCSF—A community-engaged contest to select and support a diverse “eCohort” at UCSF*; not funded, but initial feedback received from community stakeholders) First presentation made to Helen Diller Family Comprehensive Cancer Center Community Advisory Board (HDFCCC CAB) on study idea; developed into postcard project (see Fig. 2)
2019	Study name suggestions solicited from research team, with request for comments; “Eat Move Sleep” (EMOVES) study name selected Received 1-year pilot funding to assess feasibility and acceptability of online enrollment of under-represented individuals with cancer into a cancer survivors’ cohort, with focus on African American people with prostate cancer Presented to Abundant Life Faith Committee introducing EMOVES and future interview plans Interviews (Phase I) Men’s Health Committee (MCH) of the CAB voted on EMOVES logo (via Office of Community Engagement (OCE) Director email blast/Qualtrics survey)
2020	OCE helped identify dedicated EMOVES study community advisors; organized 1st community advisory call* Requested/received feedback from OCE Director and Community Education and Outreach for Urologic Cancers Chair on the idea of a study video Presented study slides and concept for video to CAB Videographer referred from OCE brought onto project to create study ad
2021	Prostate Cancer Advocate (MHC, Friends of Frank Committee; referred from OCE/CAB) brought into project (narrator for video ad) Feedback from CAB on video, feedback used to update/revise ad Interviews (Phase II) Final video ad posted on study landing page, HDFCCC YouTube, and OCE website

*EMOVES* eat move sleep study, *MHC* Men’s Health Committee, *OCE* Office of Community Engagement, *CAB* Community Advisory Board

*Study Advisors include: M. Shaw (CAB, MHC), M. Molino Saucedo (Cancer Resource Centers of Mendocino, CAB), R. Hernandez (United in Health/COVID work), W. Yu (United in Health/COVID work)

**Fig. 1:**
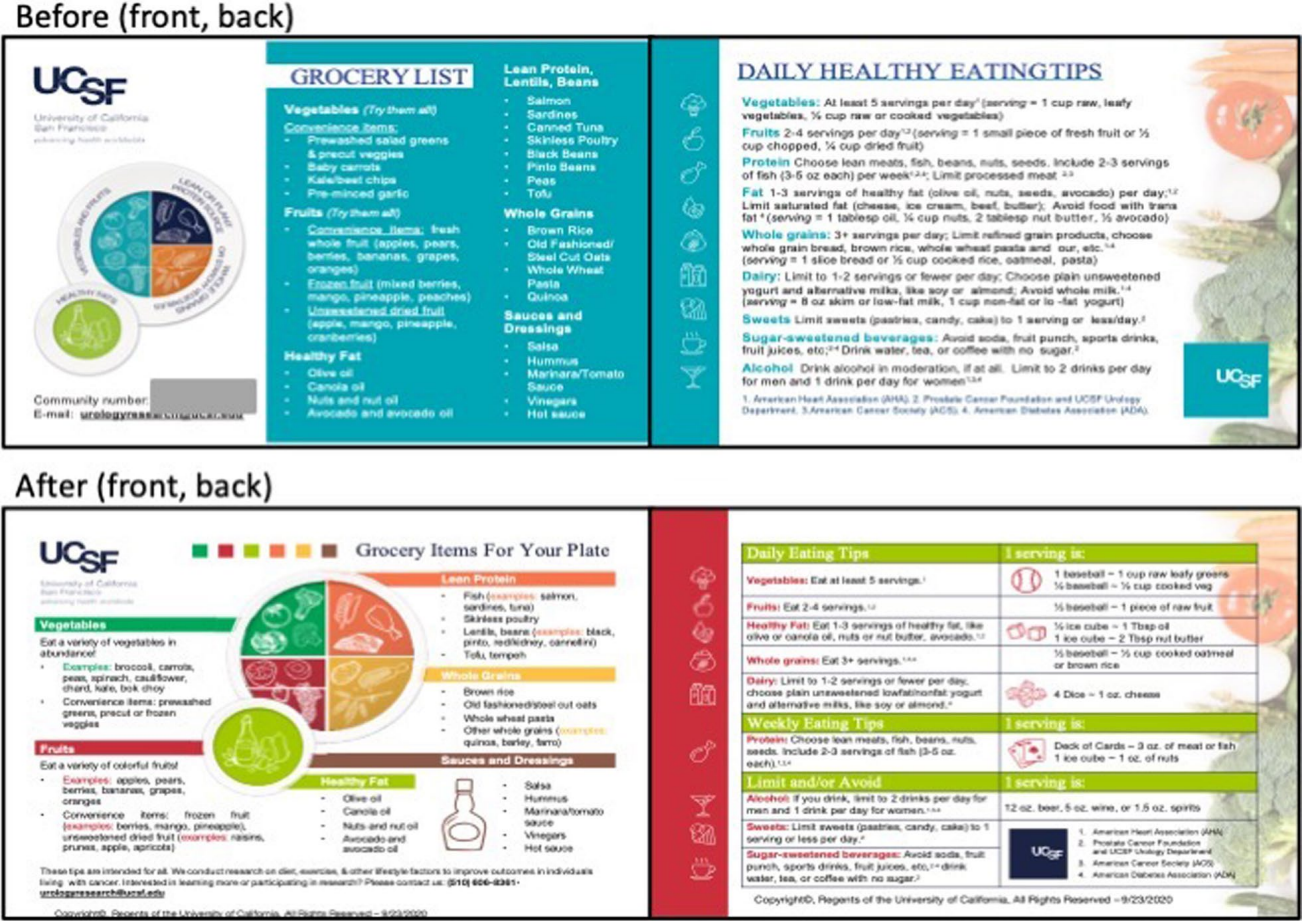
Healthy eating tips postcard development. Clockwise from top left: before (original) front, before back, after (final) front, after back) This figure shows original (November 2018) and final Healthy Eating Tips postcard (May 2019), reflecting the incorporation of Community Advisory Board feedback on visual appeal (e.g., use of earth tones instead of teal; artistic design around plate) and relatable practical content (e.g., inclusion of serving sizes using everyday items). Translated postcards into Chinese and Spanish were finished in 2021

**Fig. 2:**
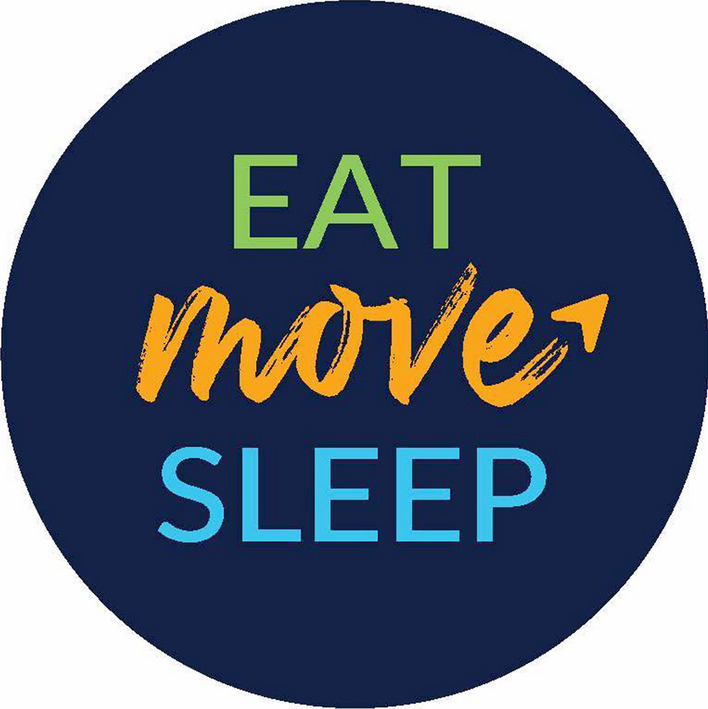
Eat move sleep study logo. Name and colors voted on by Community Advisory Board members

The first meeting with the OCE CAB in 2019 was attended by 14 individuals, representing 14 community stakeholders. We presented our broad research interests (e.g., role of diet, exercise, and other lifestyle factors on cancer survivorship), an early proposal for building a new cohort study of diverse cancer survivors, and an educational postcard focused on healthy eating tips (see below and Fig. 1). This presentation was critical to establishment of a relationship between our scientific team and community representatives. After that meeting, we engaged the OCE CAB regularly for advice and guidance (Table 2, Figs. 1 and 2).

### Feedback received, study decisions made, and additional projects pursued based on OCE, CAB, and community advisors’ input

Through the relationships that were nurtured with community stakeholders, several study decisions were made prior to and in parallel with the semi-structured user testing interviews. Examples are:

*Study Name*: The original name for the study was "Cancer and Lifestyle—a Survivorship Study (CLASS)". Through multiple requests for feedback and email voting by the OCE network, the team eventually selected "Eat Move Sleep (EMOVES)", which was well-received in the Phase I interviews.

*Study logo*: Six versions of the study logo for EMOVES were circulated via email to community members and scientific colleagues, and votes were received anonymously through Qualtrics to settle on the current logo design (Fig. 2).

*Survey topics*: Feedback from the initial OCE CAB presentation informed decisions to include questions on prayer/faith and integrative medicine. Subsequent meetings with the EMOVES community advisors clarified the need to add questions regarding financial and social needs to understand better the priorities of the population and barriers to healthy food or physical activity options.

*Additional projects pursued in response to community feedback*: Two additional projects were initiated in response to community feedback received during the development of the main study website and content—a Healthy Eating Tips postcard and a study video.

Postcard: First, in the summer of 2018, when the study engagement coordinator was attending community events, he gave out pamphlets describing open studies conducted by our team on diet and exercise for those living with cancer. Early feedback from community members suggested that we also provide a small handout (e.g., a postcard), on diet or exercise for *anyone*, since most people stopping by a health fair table are not specifically interested in cancer. Given that dietary recommendations for cancer survivors are consistent with those for cancer prevention, we created an additional educational resource; this postcard was well-received/useful as it became a regularly requested print out by CAB members and community partners for events [31, 32]. An early mockup was presented to the UCSF HDFCCC CAB in November 2018. Feedback was received and incorporated, including adding more education regarding portion size (e.g., visual tips based on common objects that correspond to portion sizes were added), dividing eating tips into categories of things to do daily versus weekly, things to avoid, and how to make the postcard more visually appealing (e.g., blue is not a color associated with food; reduce text; use more imagery) (see Fig. 1 for before and after).

The final postcard (Fig. 1) was published in English and distributed broadly at patient-facing local health symposia [UCSF/California Prostate Cancer Coalition Patient Symposium (6/8/2019) and Glad Tidings International Church of God in Christ, Faith Leaders Health Education Day, Men’s Health Symposium (7/29/19)] and presented back to the CAB in spring 2020. Given its popularity, it was translated into Chinese and Spanish, and is currently available for free download from the Urology Lifestyle and OCE Websites in three languages, and reflects a successful collaboration between the scientific team and the OCE CAB.

Video: Based on OCE and CAB feedback, and with the occurrence of the COVID-19 pandemic, many original plans with community were transitioned to online formats. In that context, our team prioritized the creation of a video ad about the study. We presented to the CAB in two phases to receive feedback on this project—first static narrative content and visuals (i.e., PowerPoint slides) were presented, followed by a draft video (Table 2). Input from the CAB led to distillation of content to clear, simple, bullet points, reduction of the video script from 384 to 273 words, replacing text and graphs from the visuals with images that evoked the study’s themes (e.g., eating, exercise), and emphasis on both the necessary time commitment for and communal goal of the study. The result was greater concordance between the audio and visual components. Furthermore, a CAB network member was nominated and subsequently invited to serve as a narrator for the ad. The final video is posted on the UCSF Cancer Center YouTube channel.

### Semi-structured user testing interview results

Ten individuals each participated in the Part I and II interviews (N = 20 total). Demographics of these individuals are summarized in Table 3. Ten of these volunteers had a history of prostate cancer, 1 had colorectal cancer, 2 had breast cancer, and 7 did not have a history of cancer and were members of the CAB community network. Participants were identified from a local African American prostate cancer support group led by a co-author (NP; N = 9), a support group led by a UCSF patient advocate (N = 3), other study referral (N = 1), and via the CAB network/events (N = 7).

**Table 3:** EMOVES interview participant demographics, by race/ethnicity

	Total	Asian	African American	Latinx	Non-Latinx white
Total	20	3	10	3	4
Gender					
Female	6	1	2	2	1
Male	14	2	8	1	3
Age					
60+	14	1	9	1	3
40–59	4	1	1	1	1
20–39	2	1	0	1	0
Cancer type					
Breast	2	0	2	0	0
Colorectal	1	0	0	0	1
Prostate	10	0	6	1	3
None	7	3	2	2	0

The purpose of Part I interviews was review of static mock-ups of the public-facing pages. The responses were positive to the bright and colorful design of the study site and logo (Fig. 1); participants reported appreciation for the racial/ethnic diversity of the people in the initial set of images for the landing page, and navigated the website mock-ups appropriately (e.g., interviewees were asked what they would do/click next after reading a specific page and to state their intentions). In response to feedback, we increased font size and decreased text density of the landing page.

The Part II interviewees reviewed revised “live” versions of the same pages, as well as the consent process and surveys. Main themes from the Part II interviews were the need for more representation of different racial ethnic/groups and gender balance in images on the landing page, survey carousel, and survey cover sheets; and requests for briefer, simpler language on the study landing and consent pages. Feedback prompted us to update imagery, and to rewrite introductory study descriptions in lay language as brief bullets (instead of narrative paragraphs) that describe study steps (landing page) and the consent form (introduction to consent page). While there were some navigational items that we could not adjust on the platform due to developer restrictions (e.g., on a standardized “Let’s Begin” page users had to click a single green button to start the survey process rather than survey images on the dashboard that appeared clickable), much of the user testing feedback was implemented, as summarized in Table 4.

**Table 4:** Semi-structured user testing interview themes

Themes	Sub-themes summary	Decision
Content	Clarity: Changes to the main study tagline of “lifestyle factors” was requested to be more specific. Requested removal of scientific jargon to explain study purpose and addition of bulleted summaries	Implemented changes
	Informative: Study instructions, summary, and page task descriptions were too wordy. Participants requested shortened summaries, bullet points and specific points of instruction before major series of task navigation	Implemented changes
	Relevance: Study summary and overview of goals were seen as redundant. Account creation required email and phone number; participants felt an email should be sufficient. IRB consent and additional study consents were seen as lengthy and signing up for the study site was perceived as a sign of consent. Activity to link a Fitbit was a recurring point of confusion for participants, as many did not own one	Partially implemented: Study summary and goals were rewritten, account sign up was simplified, and additional study sign-up pages were consolidated Changes to IRB consent were not feasible as it followed a university standard, but a summary page was provided to highlight key points While the study maintains a Fitbit option, we added text to describe this as optional. This activity was moved from prior to the study surveys to only being presented after participants completed all study surveys to minimize confusion
Usability	Interface: Placement of buttons to advance in the study and access help needed to be easier to spot and more intuitive. There was confusion regarding usage of Docusign for indicating consent; and participants were concerned that they would have to make an account and sign-in elsewhere. Survey dashboard lacked clear instructions	Implemented changes, e.g., added instructions prior to the Docusign page, added instructions about navigation and what to expect at the beginning of the first survey
Visuals	Layout: Make study logo and UCSF branding on study invite and landing page more prominent	Implemented changes
	Colors: Study color theme was favorable, but feedback was provided on the color of interactive buttons and text such that they were more easily distinguishable	Implemented changes
	Imagery: Request to diversify images to include Asian and Latinx individuals (in addition to Black adults), age-appropriate exercise, and more group-oriented visuals	Implemented changes

## Discussion

Incorporating a dedicated engagement coordinator/community health educator and collaborating with our cancer center OCE were critical to pursuing a community-based participatory research approach for developing EMOVES. Thanks to these partnerships and infrastructure, we were able to regularly engage with a CAB, identify a community advisory group for our study, obtain community input on all study elements, and complete our user testing interviews. In addition, our interactions with the community led to several lessons-learned and the collaborative development of educational resources for cancer survivors and the broader communities in our catchment area.

### Learnings from research and community partnerships

Feedback from the OCE and community advisors highlighted several crucial lessons through this iterative study development process. First, it is important to recognize community partners as experts in their communities; that expertise is the reason researchers must seek them out. Establishing the collaboration as one between research and community experts assists in deconstructing the notion that research teams or institutions are the only ones providing expertise in these partnerships. Second, investing time and attention to evolve from a simple transactional relationship based on fiscal compensation for participation/feedback to a broader dialogue around community needs that can be addressed via the resources held by research teams and/or institutions (see Table 2 and Fig. 2) has value. Third, investigators learned the value of partnering with the OCE and integrating an engagement coordinator as a permanent team member to build bridges and trust with communities. Fourth, investigators learned the importance of finding shared interests with community stakeholders, and the need for flexibility to adjust study parameters to align with the interests of community constituents. This includes keeping an open-mind and budgeting for time and resources to change direction based on community feedback. It was also critical to maintain communication and circle back to community members, so that they were aware of their value to the research process. Fifth, it is critical to recognize that community-based participatory research requires broader financial support for community partners and regular activities that nurture the institution-local stakeholder relationships, beyond the usual costs of research [33]. Availability of a funded OCE and CAB, which nurture non-transactional year-round relationships with the broader community, was the only way we could sustain community input over the duration of the EMOVES development phase (e.g., the UCSF HDFCCC OCE supported honorariums for our community advisory group and provided backup support for the dedicated engagement coordinator). For researchers who do not have such an institutional resource, we recommend identifying and partnering with local organizations (e.g., community clinics) that are able to host ongoing partnerships with community health stakeholders and allowing for additional ramp-up time to apply for grant support that includes costs for the community partners [34]. Additionally, our team has discussed ways researchers can engage in non-transactional relationship-building with the EMOVES advisors and larger CAB network and constituency, year-round, independent of the study status and activities (e.g., sharing information in lay language on scientific news related to health behaviors and cancer survivorship via periodic emails or Dropbox file sharing).

For community partners, there was a learning curve to understand the role and timeline of grant funding that affected our scientific team’s ability to execute ideas (e.g., while we liked the idea of producing more educational materials, doing so was unfunded). The dedicated EMOVES community advisors have come to understand the cycle of National Institutes of Health (NIH) grant applications.

While we report here on the journey of sustained engagement with community partners to develop the infrastructure for a novel cohort study of cancer survivors, there are several limitations to consider. In response to the COVID-19 pandemic, several original plans for community engagement and recruitment (e.g., having a physical presence at Black churches or barbershops) had to be adjusted to online formats, which limited contact with people who were not as comfortable with technology. Sometimes, community feedback included requests for materials that were beyond our budget or current bandwidth (e.g., culturally tailored diet booklets for different populations (e.g., one geared for Latinx persons), making a cookbook, providing local shopping guides, etc.). Interest in these ideas were noted for future initiatives.

In conclusion, our team successfully engaged with the community to guide pilot work for developing a future cohort study of diverse cancer survivors. This work was enabled by the dedicated efforts of a community engagement coordinator and strong partnerships fostered through cancer center infrastructure for community outreach and engagement. The long-term goal of EMOVES is to unravel interactions among sociodemographic and social factors (e.g., financial toxicity), diet, physical activity, sleep habits, and their relationship over time to quality of life and clinical outcomes (e.g., receipt of guideline-concordant care, risk of cancer progression, mortality). Next steps for this research include applications for further grant funding to expand recruitment to under-represented populations using the California Cancer Registry and support long-term medical record follow-up.

### Supplementary Information

**Additional file 1. **GRIPP2 Long Form checklist.

## Data Availability

Community Advisory feedback and focus group interview questions are available in Table 4 and Supplemental Methods.
