# Supplementary material for: Engaging communities to inform the development of a diverse cohort of cancer survivors: formative research for the eat move sleep study (EMOVES)
Source: Res Involv Engagem. 2023 Dec 11;9:117. doi: 10.1186/s40900-023-00529-z (PMC10712178; doi:10.1186/s40900-023-00529-z)
Supplement: Supplementary file 1 — Additional file 1. GRIPP2 Long Form checklist. [file 40900_2023_529_MOESM1_ESM.docx]

**Additional file 1: Methods**

**Eat Move Sleep Study Prebuild and Build User Testing Guide**

Introduction: Thank you so much for coming today! As you may know, we’re working to build an online medical research study and we need your feedback. Our goal today is to improve the product and while we may see issues, I want to assure you that we’re testing the product, NOT YOU. We would like our product to work for you.

I’m going to ask you to do a couple of things that may seem weird at first. I’d like to ask you to think aloud as we work together. It will feel a little awkward, but here, let me demonstrate. I’m going to double click on this icon to open up photo booth. This will allow us to record this session. Are you OK with recording? Our goal is only to make taking notes easier.

Talk aloud. Don’t be afraid to ask questions but I may not answer all because we’re interested in what you might think.

Couple of simple questions to get you used to talking…

Do you use the Internet or email, at least occasionally? Do you access the Internet on a cell phone, tablet, or other mobile handheld device, at least occasionally?

Overall, how often do you use the internet — several times a day, once a day, at least once a week or less often?

What are some common things you’ve used a computer, a tablet, or your cell phone to do?

What was the last thing you did on the internet?

Have you ever participated in medical research before?

Tasks

Get Online

I’ve opened a [prototype of our product](https://invis.io/MFQY1VQ2C39#/368051294_Overview_Page_-1-). This is a work in progress. We won’t be able to actually enter any of your information, so it won’t work exactly like the product. Here’s an email with an invitation to our research study. Can you take a quick look:

1. **Decide to Participate & Sign Up**
2. What do you think you can do on this page? What would you do next? Where would you go to find out more information?
3. What do you think this study is about?
4. Do you want to participate? If you wanted to participate, what do you think the next step is? Could you go ahead and do that?
5. What do you think you would need to provide in order to move forward? Could you go ahead and try that?
6. Imagine I’m not here, what would you typically do next?
7. How do you feel about sharing your personal information to sign up?
8. **HIPAA Authorization**
9. What do you think you are being asked to do? Why are you being asked to do this?
10. What are the next steps and what do you think you’ll need to do? Ok let’s try that…
11. How would you move ahead? Can you go ahead and try that?
12. How well do you think you’ve done? What else do you think you’ll need to do?
13. What do you think has happened? Anything you think for you to do now? What else do you think you can do? How many options do you think you have?
14. Have you used DocuSign before?
15. Do you feel comfortable using DocuSign?
16. **Fitbit Connection**
17. How do you think you could finish up? What options do you have?
18. What else could you do on the site?
19. Do you own and or are familiar with Fitbit?
20. **Finding the resources page**
21. (Home page) what else do you think you can do on this page?
22. If you wanted to learn more about lifestyle changes for cancer survivors, what you would do next?
23. Are you interested in these resources? Do you currently have health resources you use? How do these resources compare?
24. What did you expect from a “resources” page associated with this study?
25. **Look and feel**
26. Tell me about the images/pictures you saw. How did they meet or miss your expectations?
27. If there was a theme for this study, what color would it be? What color do you most associate with this study?
28. Was the text presented in a way that was understandable and easy to read?
29. **Survey landing page (build display)**
30. What can you do on this page?
31. Where would you start first?
32. Is the number of tasks and time required for each clear?
33. Can you leave and return to this page and feel comfortable to start where you left?

Thank you this has been great. We’ve learned so much!

GRIPP2 long form

| Section and topic | Item | Reported on page No |
| --- | --- | --- |
| Section 1: Abstract of paper | | |
| 1a: Aim | Report the aim of the study | 2 |
| 1b: Methods | Describe the methods used by which patients and the public were involved | 2 |
| 1c: Results | Report the impacts and outcomes of PPI in the study | 2-3 |
| 1d:Conclusions | Summarize the main conclusions of the study | 3 |
| 1e: Keywords | Include PPI, “patient and public involvement,” or alternative terms as keywords | 2 |
| Section 2: Background to paper | | |
| 2a: Definition | Report the definition of PPI used in the study and how it links to comparable studies | 4 |
| 2b: Theoretical underpinnings | Report the theoretical rationale and any theoretical influences relating to PPI in the study | 4 |
| 2c: Concepts and theory development | Report any conceptual or theoretical models, or influences, used in the study | 4 |
| Section 3: Aims of paper | | |
| 3: Aim | Report the aim of the study | 4 |
| Section 4: Methods of paper | | |
| 4a: Design | Provide a clear description of methods by which patients and the public were involved | 6-9 |
| 4b: People involved | Provide a description of patients, carers, and the public involved with the PPI activity in the study | 5-9 |
| 4c: Stages of involvement | Report on how PPI is used at different stages of the study | 5-9 |
| 4d: Level or nature of involvement | Report the level or nature of PPI used at various stages of the study | 7-9 |
| Section 5: Capture or measurement of PPI impact | | |
| 5a: Qualitative evidence of impact | If applicable, report the methods used to qualitatively explore the impact of PPI in the study | N/A |
| 5b: Quantitative evidence of impact | If applicable, report the methods used to quantitatively measure or assess the impact of PPI | N/A |
| 5c: Robustness of measure | If applicable, report the rigor of the method used to capture or measure the impact of PPI | 6-9 |
| Section 6: Economic assessment | | |
| 6: Economic assessment | If applicable, report the method used for an economic assessment of PPI | N/A |
| Section 7: Study results | | |
| 7a: Outcomes of PPI | Report the results of PPI in the study, including both positive and negative outcomes | 10-14 |
| 7b: Impacts of PPI | Report the positive and negative impacts that PPI has had on the research, the individuals involved (including patients and researchers), and wider impacts | 10-14 |
| 7c: Context of PPI | Report the influence of any contextual factors that enabled or hindered the process or impact of PPI | 10-11 |
| 7d: Process of PPI | Report the influence of any process factors, that enabled or hindered the impact of PPI | 11-14 |
| 7ei: Theory development | Report any conceptual or theoretical development in PPI that have emerged | 11-14 |
| 7eii: Theory development | Report evaluation of theoretical models, if any | N/A |
| 7f: Measurement | If applicable, report all aspects of instrument development and testing (e.g., validity, reliability, feasibility, acceptability, responsiveness, interpretability, appropriateness, precision) | 13-14 |
| 7 g: Economic assessment | Report any information on the costs or benefit of PPI | 7 |
| Section 8: Discussion and conclusions | | |
| 8a: Outcomes | Comment on how PPI influenced the study overall. Describe positive and negative effects | 15-17 |
| 8b: Impacts | Comment on the different impacts of PPI identified in this study and how they contribute to new knowledge | 15-17 |
| 8c: Definition | Comment on the definition of PPI used (reported in the Background section) and whether or not you would suggest any changes | 15 |
| 8d: Theoretical underpinnings | Comment on any way your study adds to the theoretical development of PPI | 17 |
| 8e: Context | Comment on how context factors influenced PPI in the study | 16 |
| 8f: Process | Comment on how process factors influenced PPI in the study | 16 |
| 8 g: Measurement and capture of PPI impact | If applicable, comment on how well PPI impact was evaluated or measured in the study | N/A |
| 8 h: Economic assessment | If applicable, discuss any aspects of the economic cost or benefit of PPI, particularly any suggestions for future economic modelling. | N/A |
| 8i: Reflections/critical perspective | Comment critically on the study, reflecting on the things that went well and those that did not, so that others can learn from this study | 17 |
